# Complement Activation in the Central Nervous System: A Biophysical Model for Immune Dysregulation in the Disease State

**DOI:** 10.3389/fnmol.2021.620090

**Published:** 2021-03-04

**Authors:** Nicholas Peoples, Candace Strang

**Affiliations:** ^1^Baylor College of Medicine, Houston, TX, United States; ^2^IPPIN Biomarkers, Inc., Arlington, MA, United States

**Keywords:** CR1, factor H, C1 inhibitor, complement, neuroimmune, Alzheimer's disease, schizophrenia

## Abstract

Complement, a feature of the innate immune system that targets pathogens for phagocytic clearance and promotes inflammation, is tightly regulated to prevent damage to host tissue. This regulation is paramount in the central nervous system (CNS) since complement proteins degrade neuronal synapses during development, homeostasis, and neurodegeneration. We propose that dysregulated complement, particularly C1 or C3b, may errantly target synapses for immune-mediated clearance, therefore highlighting regulatory failure as a major potential mediator of neurological disease. First, we explore the mechanics of molecular neuroimmune relationships for the regulatory proteins: Complement Receptor 1, C1-Inhibitor, Factor H, and the CUB-sushi multiple domain family. We propose that biophysical and chemical principles offer clues for understanding mechanisms of dysregulation. Second, we describe anticipated effects to CNS disease processes (particularly Alzheimer's Disease) and nest our ideas within existing basic science, clinical, and epidemiological findings. Finally, we illustrate how the concepts presented within this manuscript provoke new ways of approaching age-old neurodegenerative processes. Every component of this model is testable by straightforward experimentation and highlights the untapped potential of complement dysregulation as a driver of CNS disease. This includes a putative role for complement-based neurotherapeutic agents and companion biomarkers.

## Introduction

**Figure 1:**
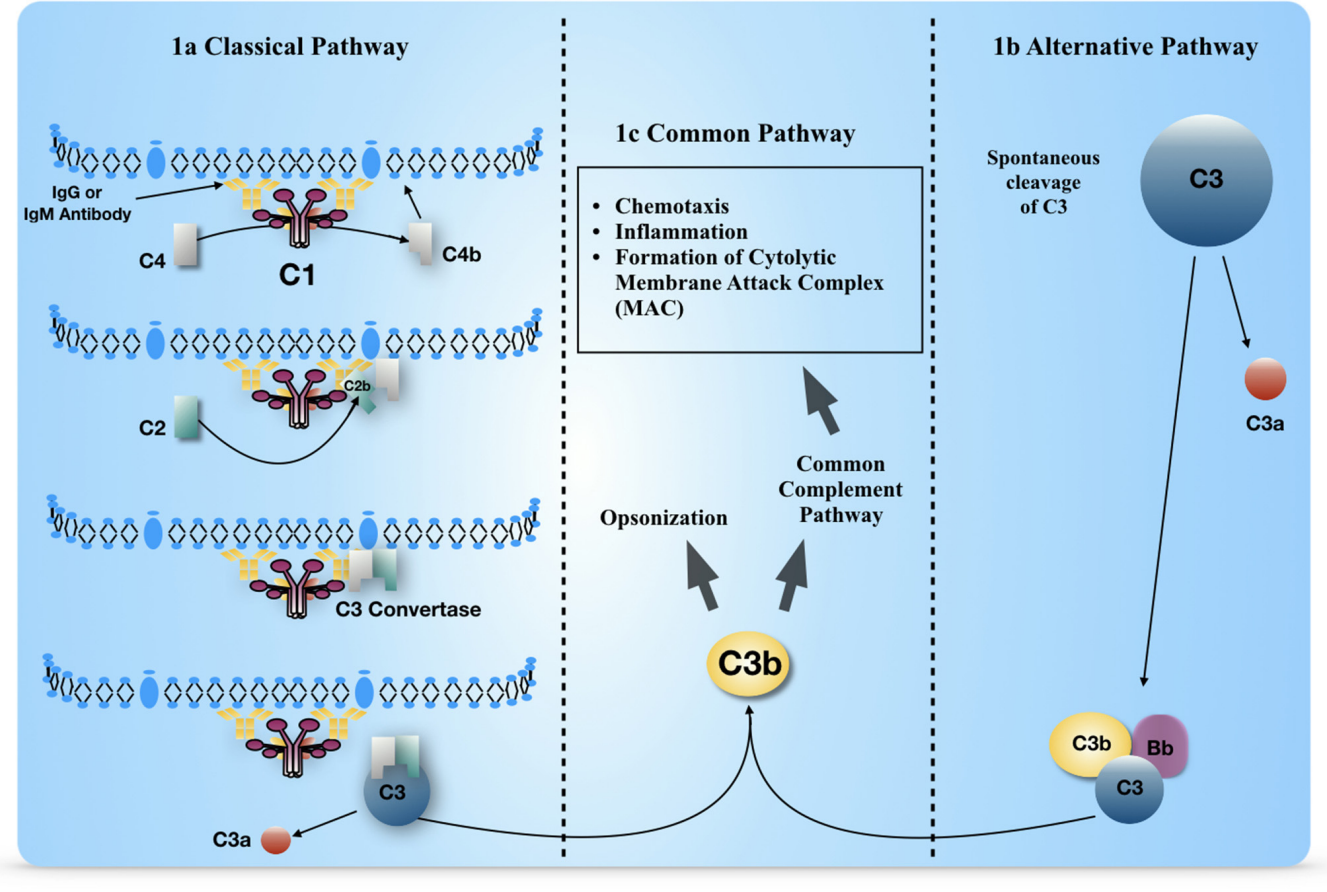
Elements of the classical and alternative complement pathways, separated by the unique and common aspects. **(a)** Early steps of the classical pathway, to show activation and generation of C3 convertase enzymatic activity. C4b (soluble) is converted to C4b (membrane bound) via a thiol ester transesterification reaction (not shown in figure). **(b)** Early steps of the alternative pathway to show activation and generation of C3 convertase enzymatic activity. **(c)** Common aspects of complement pathway activation that result in macrophage and neutrophil recruitment and formation of the membrane attack complex, a cytolytic pore on the pathogen surface.

The basic features of complement, with three separate activation processes that converge into a common pathway for immune signaling and pathogen clearance, are well-understood (Merle et al., 2015a,b; Murphy and Weaver, 2016). The classical complement pathway (CCP) identifies immune complexes of IgG or IgM and certain other macromolecules independently of antibody (Figure 1a). The alternative pathway activates in an antibody-independent manner when Complement C3 spontaneously hydrolyzes into C3 (H_2_0) and combines with complement factor Bb (Figure 1b). Activation of either pathway triggers a cascade of enzyme reactions, chemotactic signaling to nearby immune cells, and tagging the pathogen for removal by immune cell-mediated uptake (Figure 1c). The rapid, exponential activation of this defense system provides immediate protection to the host, but also the possibility of great damage if the regulatory brakes fail. The first step of pathogen recognition is the only molecular recognition event for definitive pathogen identification. All subsequent steps in complement activation occur in a programmed fashion. Therefore, several regulatory elements limit activation of complement to ensure complement only targets unwanted material and not host tissue.

Until 1998, a mistaken assumption pervaded the scientific community that the brain did not have its own immunity due to protection behind the blood brain barrier (BBB). This erroneous assumption was exposed upon demonstration of MHC I cell surface markers for self in the brain by Carla Shatz and coworkers (Corriveau et al., 1998). Shortly after, several groups demonstrated evidence of complement protein synthesis in the brain (Terai et al., 1997; Singhrao et al., 1999). Baseline CNS complement levels are made by microglia, and, under stressful conditions (e.g., oxidative stress and inflammation), can be made by neurons and astrocytes (Kolev et al., 2009). Over the next two decades researchers detailed how the CCP is used to selectively remove unused or damaged synapses (Stevens et al., 2007; Chu et al., 2010; Sekar et al., 2016) in a process called synaptic pruning. Remarkably, only a fraction of synapses is removed at any one time in a directed fashion that is not fully understood. Given this specialized and discriminating function, tight regulation of complement is paramount to ensure precision removal of only unwanted synapses.

**Figure 2:**
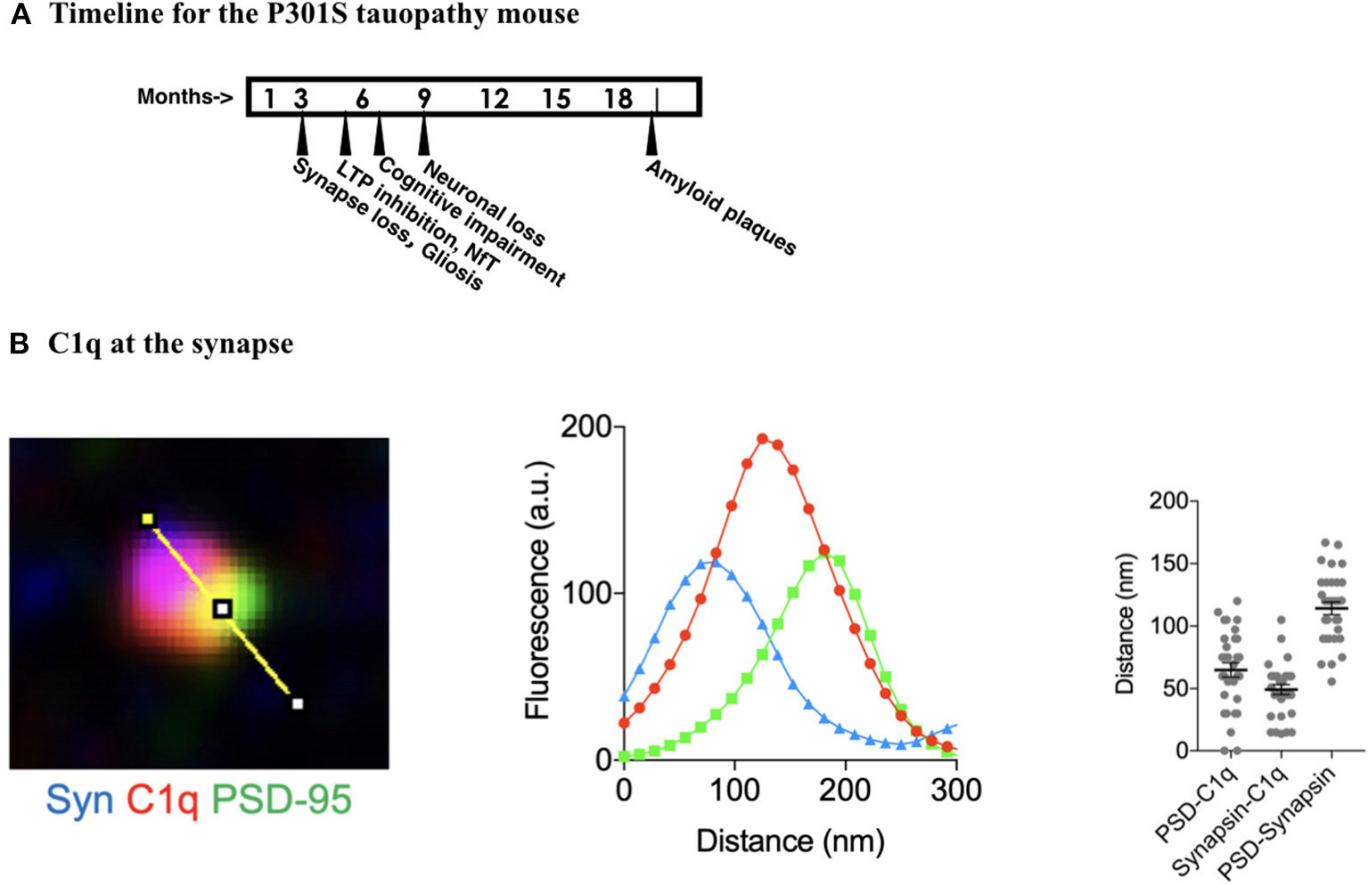
Complement activity at the synapse in the brain of AD tauopathy mice. **(A)** Timeline for Alzheimer's Disease signs and symptoms to develop in P301S mouse. This mouse is analogous to a hereditary form of AD with a mutation in the tau gene that is a conversion from proline to serine at amino acid position 301. Timeline information collected from www.Alzform.org. **(B)** Measurements taken from micrographs of the synaptic region of the P301S mouse hippocampal CA1 region where neuronal structure is disrupted, at nine months. Data from Dejanovic et al. (2018) with permission. Fluorescence recordings from Synapsin at the presynaptic membrane, C1q, and PSD95 at the post-synaptic membrane. Analogous samples from the hippocampal CA1 region of mice without the P301S addition showed C1q staining that was barely detectable, indicating that the C1q location and increased density was due to the AD like pathology in the P301S mice.

There are two distinctive features of CNS complement. The first is that complement can be activated to remove select parts of host cells as a natural aspect of development. In neuronal development, older cells are sometimes removed in anatomical development with changes with maturity to an adult pattern. Complement participates in this removal process (Stevens et al., 2007, 2018; Coulthard et al., 2018; Presumey et al., 2018). The second feature is the elimination of neuronal synapses thought to be damaged in neurodegenerative diseases such as AD. In AD, it has long been known that complement binds to neurofibrillary tangles (NfT) and amyloid plaques (Eikelenboom et al., 1989; Rogers et al., 1992; Shen et al., 2001; Tacnet-Delorme et al., 2001; Hong et al., 2016). More recent studies, using high-resolution microscopy techniques, have yielded a remarkable picture of complement proteins at the synapse (Dejanovic et al., 2018; Litvinchuk et al., 2018) (Figure 2). In AD, reduced synaptic density corresponds to altered synaptic plasticity (Sheng et al., 2012), such as impaired long-term potentiation (LTP) and enhanced long-term depression (LTD), leading to cognitive impairment. Complement activity participates in synapse removal.

CNS complement is capable of clearing fragments of host cells and synaptic connections in the brain, both in development and in disease. This underscores a clear concept for neurological disease. We therefore hypothesize that overactive or underactive (i.e., dysregulated) complement activity may create a pattern of abnormal circuitry in the brain. If the level of synaptic clearance is too low, excess synaptic connections may lead to aberrant activity (e.g., hyperactivity, seizures, lack of definitive signaling circuitry). If the level of synaptic clearance is too high, insufficient circuitry remains and neurodegenerative conditions are created. Moreover, complement directly binds NfT and amyloid, underscoring a link to the progression in AD. Other publications have elegantly described several aspects of complement activity in the CNS, including location, microglial interactions, and pharmacologic inhibition (Veerhuis et al., 2011; Shen et al., 2013; Morgan, 2018; Bohlen et al., 2019; Wilton et al., 2019). This paper highlights a critical missing piece, calling attention to regulatory failure as a major potential mediator of CNS disease. First, we propose pathogenic variants of regulatory complement proteins (Factor H, Complement Receptor 1) may result in excess C3b and failure to protect host tissue. Second, we illustrate how simultaneous activation of the complement and contact pathways may overwhelm C1-inhibitor, producing potentially catastrophic prolonged activation of the entire complement pathway. Finally, we suggest putative roles for CUB Sushi multiple domain proteins as complement control proteins and raise the intriguing question of whether their unique domain structure could imply participation in cell-signaling complexes at the post-synaptic density. Collectively, this model offers a unique perspective on neuroimmune relationships. Each component spurs new directions for interdisciplinary, high-impact research and underscores the potential of complement-based neurotherapeutic approaches.

## Disease Mutations of Regulators of Complement Activation Proteins Lead to Increased Complement Activity

### Excess C3b Can Damage Host Tissue

C3b is the foremost signaling fragment that provides the “EAT ME” opsonization marker recognized by surface receptors on phagocytic immune cells. Soluble C3b remains capable of generating phagocytic complement activity as it diffuses away from the original pathogenic target and it has no special recognition capacity to distinguish a pathogen vs. self-cell. It is therefore critical to limit the number of C3b fragments that are generated, either by inhibiting the C3 convertase or catalyzing C3b into iC3b (inactivated C3b).

The Regulation of Complement Activation (RCA) proteins each play a role in limiting the level of biologically active C3b. This family of proteins are encoded in a gene cluster on chromosome 1 and are characterized by a quaternary structure that are repeats of the so-called “Sushi” domain, a conserved five-stranded β-sheet domain of ~ 60 amino acids (Furtado et al., 2008; Wu et al., 2009; Forneris et al., 2016; Xue et al., 2016). They fall into two categories: soluble or membrane bound. Soluble regulators are critical for limiting the amount of C3b generated; membrane-bound regulators remove errantly bound C3b from the surface of host cells. The ensuing section will discuss Factor H, a soluble RCA protein, and the section following that will consider Complement Receptor 1, a membrane bound RCA protein, in great detail.

### Mutations in Factor H Can Create Excess C3b

**Figure 3:**
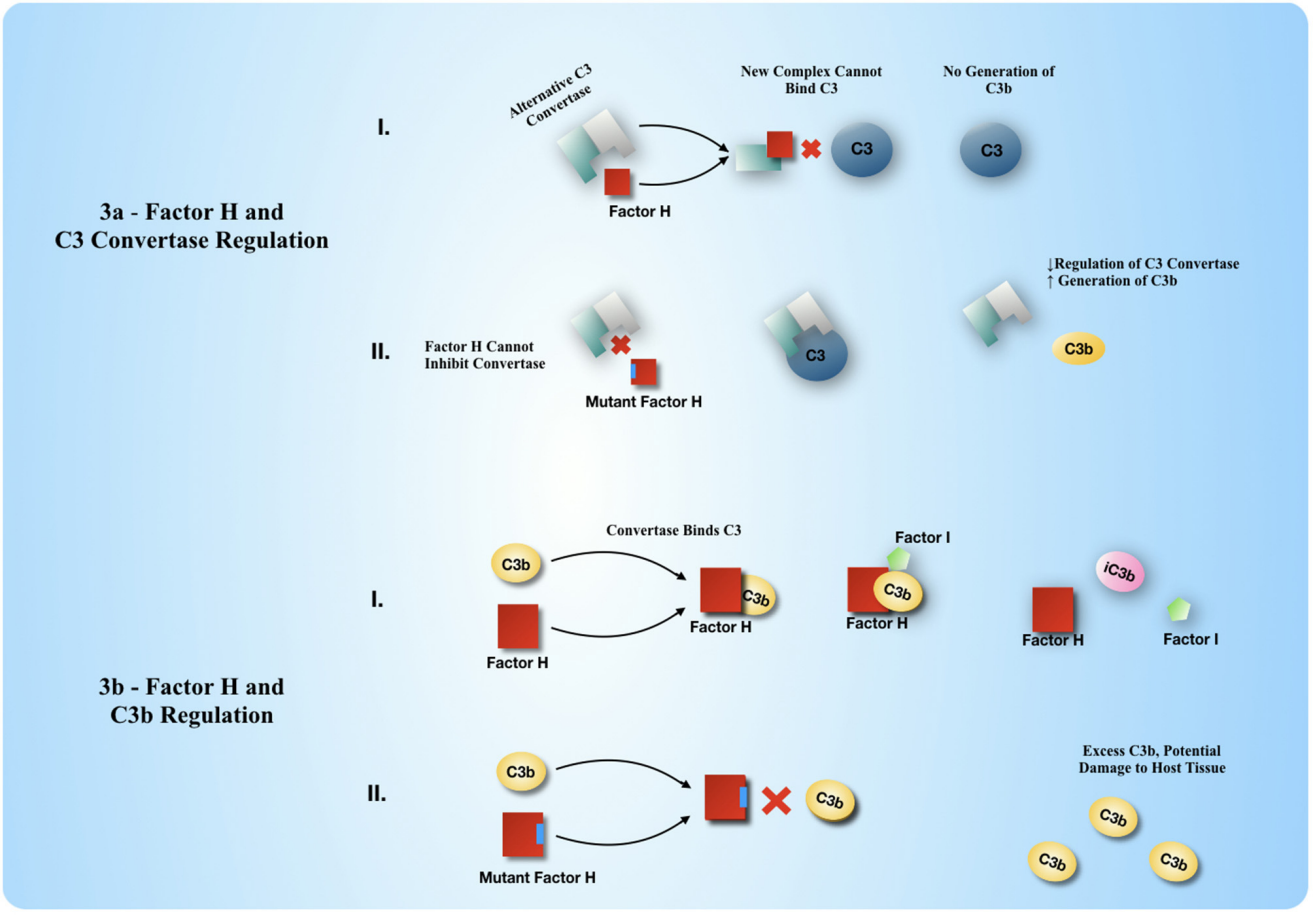
Factor H regulates the generation of C3b and the inactivation of C3b. **(a)** Factor H regulates the generation of C3b by active disassembly of the C3 convertase enzyme. Factor H displaces the Bb binding partner to C3b to make a new complex with C3b that is Factor H: C3b, hence ceasing further generation of C3b. **(b)** Factor H regulates the level of C3b by participating in further enzymatic degradation of C3b to iC3b and other smaller protein fragments. Factor H acts as a cofactor to Factor I to bring C3b and Factor I together so that factor I may degrade C3b further through proteolysis. iC3b and smaller fragments of C3 can no longer be a part of any C3 convertase assembly, either on a cell surface or as a soluble enzyme in solution.

The soluble RCA proteins, such as Factor H, are diffusible factors that serve to regulate both soluble and cell-bound C3 convertase enzymes of complement. Mutations in Factor H handicap its ability to dissociate the alternative pathway C3 convertase (Figure 3) and/or directly cleave C3b to iC3b with the assistance of the soluble protease factor I. The result is excess C3b and limited conversion to iC3b which may override host protection mechanisms, aberrantly allowing C3b to signal for host tissue to be cleared. This excess C3b can be soluble, diffusible, and a ligand for any of the cell-bound RCA proteins. This process is documented in the periphery, where Factor H mutations are associated with eye and kidney disease (Ferreira et al., 2010). CNS tissue is densely packed (where diffusing C3b could easily land on an improper target), and Factor H is a risk factor for AD (Zhang et al., 2016) and schizophrenia (Boyajyan et al., 2010), so the concept of CNS-centered disease is well within reason.

Proteins unique to the classical complement pathway are risk factors for AD (Jones et al., 2010). Specifically, MHC III proteins which directly impact the formation and disassembly of the various C3 convertases (Factor B, C2, C4) are risk factors for AD (Alzheimer Forum Chromosomal location for AD risk genes) and schizophrenia (Sekar et al., 2016; Woo et al., 2020). Since both formation (C2, Factor B) and clearance (factor H) of the C3 convertase are processes influenced by disease risk factors, it is reasonable that C3b may be a pivot point in CNS complement biology and its level dictates the effectiveness of this immune clearance function. The classical complement pathway in the CNS proceeds at least up to the point of C3 cleavage. Immunohistochemical studies have not yet progressed to the level of extensive complement factor analysis, however, so we suggest this is a valuable area for future research.

### Complement Receptor 1 Is Strongly Associated With Alzheimer's Disease and Mutations May Lead to Aberrant C3b Deposition and Clearance of Neurons

Complement Receptor 1 (CR1), a membrane-bound RCA protein, is located on neurons (Lian and Zheng, 2016) and cells in their immediate vicinity (Lucin and Wyss-Coray, 2009). CR1 is among the top 10 risk factors for AD (Lambert et al., 2009; Jun et al., 2010; Allen et al., 2015; Kunkle et al., 2019). CR1 acts as a cofactor to Factor I to cleave C3b that is on or near to the cell surface of a host cell. This C3b can be diffusing C3b or C3b that is covalently attached to a cell surface moiety. C3b is inactivated and made Convertase incompetent by conversion to iC3b (Figure 5). Thus, CR1 serves to limit the extent of complement activity by the prevention of C3b binding to nearby self-cells so they are not tagged for immune clearance. We hypothesize that mutations in CR1 handicap its ability to deactivate C3b, and the end result is loss of protection to essential brain cells from immune-mediated clearance. The normal number of CR1 receptors on a neuron is unknown, but cell surface CR1 has been characterized on blood plasma cells: only 100-1200 copies on red blood cells and ~20,00–50,000 on nucleated cells of the immune system (Martin, 2007), so even a small disruption of CR1 functional capacity or structural stability could have significant deleterious effect. In contrast, a more abundant receptor such as CR3 is present on the cell surface in 100,000–200,000 copies (Abbas et al., 2019).

**Figure 4:**
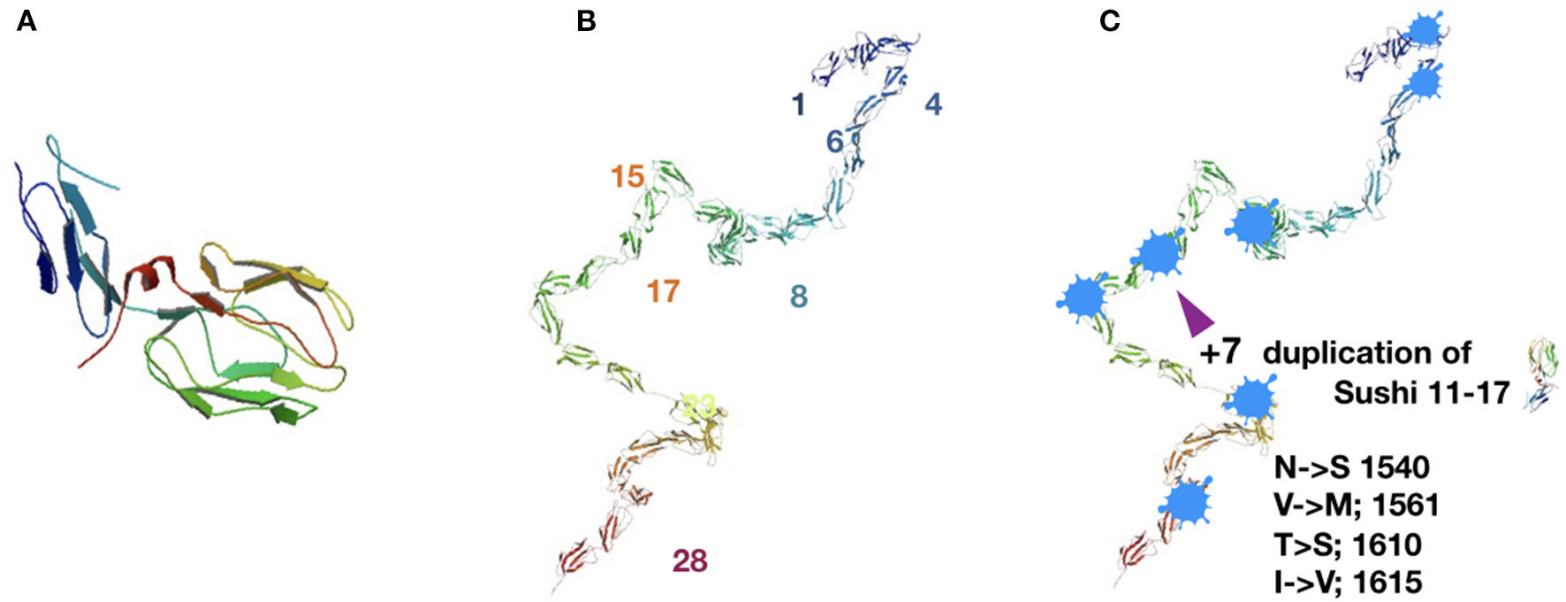
Complement receptor 1 biology with the basic Sushi subunit domain repeat and major and minor alleles of the gene. **(A)** Single Sushi domain composed of ~60 amino acids in beta sheet array, two stranded beta sheet facing three strand beta sheet, anti-parallel alignment. Major allele (left) in 83% of the population. Structure is an extracellular domain of 30 consecutive Sushi domains. Structure PDB 2Q7S, from the laboratory of SJ Perkins at UCL (Furtado et al., 2008). **(B)** Minor allele (right) in 11% of the population. The transmembrane domain (not shown) and basic Sushi domain subunit are unchanged. There is a seven Sushi repeat of Sushi domains 11–17, that is located directly adjacent to the original location of the duplicated Sushi domains. The binding site for C3b is located at domains 15–17, so this repeated segment of Sushi domains 11–17 includes a second binding site for C3b. **(C)** Inkblot; regions of predicted intrinsic disorder within the CR1 amino acid sequence. These are short regions of amino acid repeats or regions mixed with charged amino acids and hydrophobic amino acids in close proximity that create ambiguity about preference for hydrophilic environment or hydrophobic surroundings instead. Prediction programs used were: PONDR developed by Romero, Li, Dunker, Obradovic and Garner, www.pondr.com, described in Xue et al. (2010). IUPRED, www.iupred2a.elite.hu, described in Dosztányi et al. (2005).

There are multiple mechanisms to explain how CR1 mutations might lead to the loss of host cell protection. The simplest is decreased stability in the mutant protein that results in reduced expression or functionality of CR1 on the cell surface. Normal CR1 moves throughout the membrane and patrols the cell surface (Petty et al., 1980). There may be poor tolerance for a mutant CR1 with limited mobility or decreased cell surface density, resulting in C3b levels that are unchecked by inactivation to iC3b and cell – surface removal. Point mutations of CR1 associated with the Knops blood group are known to confer increased risk for AD (Sandri et al., 2017) so the concept is within reason. These mutations fall in clusters near the membrane surface (Figure 4). The structural requirements within this region are particularly strict without much room for amino acid side chain variation (the mutations in Figure 4 are each quite conservative). This may relate to interactions with the membrane, ligands, or specific parameters in folding or trafficking that prevent a stable cell surface receptor. The associated risk for AD is small, and these mutations do not account for the increased frequency that is reported in genome wide association studies (GWAS) for AD risk (Lambert et al., 2009; Jun et al., 2010; Allen et al., 2015; Kunkle et al., 2019).

**Figure 5:**
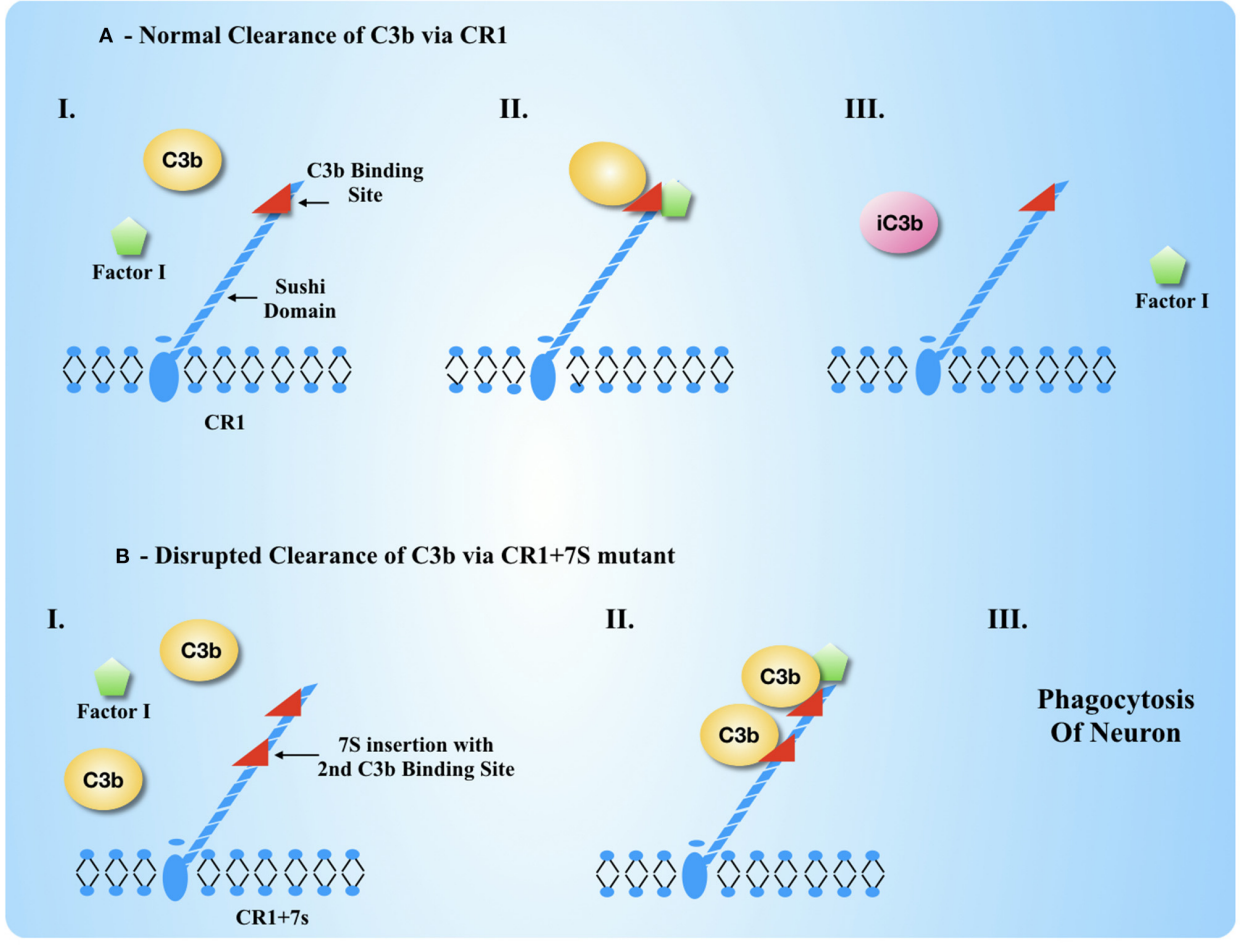
CR1 Biology with anticipated changes due to duplicated sushi domains in CR1 biology normally and under dysregulation. **(A)** Under normal circumstances, CR1 acts as a cofactor to Factor I to cleave C3b into iC3b, which is released so that another C3b may be bound and cleaved. **(B)** Mutant CR1 with a second C3b binding site (CR1+7S) is unable to release the C3b dimer because the dimer may bind more tightly. The result is that the cell with this CR1+7S on the surface is incorrectly tagged for phagocytosis or endocytosis.

A CR1 mutant that carries significant AD risk (two-fold increase) contains a seven Sushi domain insertion (11–17) and an extra C3b binding site. We will refer to this mutant as CR1+7S^1^. CR1+7S is present in ~10% of the general population (Brouwers et al., 2012; Hazrati et al., 2012; Almedia et al., 2018), and, under laboratory conditions, has increased affinity for C3b (Fonseca et al., 2016). Perkins et al. found that C3b dimers bind to CR1 with increased affinity, and that dimers and higher multimers of C3b may form in the presence of Zinc (Nan et al., 2013). Prior to the report from the Perkins laboratory, C3b dimers formed from oxidation reactions were known and studied. Elevated Zinc levels and an oxidative environment are known factors in AD (Yan et al., 2013; Bhat et al., 2015; Adlard and Bush, 2018; Zsolt et al., 2020), so C3b dimers are an expected feature of complement biology under the stressful conditions that characterize early neurodegeneration. Should a C3b dimer bind CR1+7S, it may well-bind with greater affinity than C3b in the traditional CR1: C3b complex. The original purpose to inactivate C3b and remove it from the cell surface by internalization may then be defeated. CR1 is known to respond differently depending on the ligand that is bound to it (Java et al., 2015). While C3b or iC3b-bound CR1 is internalized, CR1 bound to C3b that is itself bound to immune complexes is *not* internalized. Consequently, this complex remains on the cell-surface as an opsonization marker. Cell surface C3b or iC3b provide an “EAT ME” opsonization marker on the host self-cell which expresses the CR1 for its own protection. If the complex of a C3b dimer and CR1+7S is not internalized, the complex would then errantly tag a host cell for immune-mediated destruction. This highlights a clear mechanism for potential neurodegeneration (Figure 5).

Existing experimental evidence fits well with this concept. The Rogaeva and Broeckhoven laboratories took human tissue samples with and without the 7-Sushi CR1 insertion and analyzed them for CR1 and the intracellular protein ERGIC-53 (Hazrati et al., 2012). Neurons with a 30-Sushi domain CR1^+^ showed a fusiform architecture and co-localization of CR1 with ERGIC-53, indicating normal recycling events for Type 1 membrane proteins such as CR1. In the AD tissue samples, C3 was identified in a diffuse pattern within the neurons as well as on amyloid and tangle aggregates. The 37-Sushi domain CR1^+^ neurons were found to have a vesicular pattern, without ERGIC-53 colocalization or C3 staining in the same neurons, even though C3 on amyloid and tangles was noted. The vesicular pattern was considered to be a lysosomal location for the larger CR1 receptor. We interpret this finding as soft support for the outcome that the larger CR1 molecules endure the fate of phagocytoses by microglia rather than internalization, since it is expected that the 37-Sushi CR1 with an additional C3b binding site retains C3b binding capacity. As expected, both CR1 receptors have increased expression in the AD tissues. However, the CR1/CR1+7 S ratio (~4.5:1, as predicted from the relative prevalence for these two genes, and confirmed in this study) is not maintained, with a larger AD-related increase found for the 37-Sushi CR1 rather than the 30-Sushi CR1 (AD ratio ~2 CR1:1 CR+7S), for an unknown reason. We suggest this observation might be followed with further molecular dissection of these cells to further understand what complexes are present and what different cellular processes are involved.

### The CR1+7S Variant May Complex With Additional Binding Partners Which Influence the Risk of Alzheimer's Disease

In this section, we will outline a structure-derived model which shows how such a cell surface CR1+7S might attract additional binding partners which influence AD risk. It is long-established that CR1: C3b: Factor I briefly form a trimeric association for the inactivation of C3b. We suggest the CR1+7S variant with bound C3b dimer (C3b_2_) could form a more stable association of CR1: C3b: C3b: factor I, with factor I retained in complex based on an altered kinetic profile for this reaction. This stable complex is rich in complex protein structure and may provide a scaffold for a larger protein assembly.

The first proposed binding partner is ApoE, which fits nicely with findings from population-level data. As already mentioned, CR1 is among the strongest known AD risk factors. Similarly, the ε4 allele of ApoE is the highest risk gene for sporadic AD. The genes for ApoE and CR1+7S both confer AD risk separately. When the genes are present together, however, the risk is increased beyond what would be expected if the genes were operating as AD risk factors independently of each other. This implies an interaction between CR1 and ApoE. Our complex suggests a mechanism by which this may happen. The Factor I structure includes two LDL receptor A type domains, and ApoE is capable of binding this type of domain (Yamamoto et al., 2008; Guttman et al., 2010; Nilsson et al., 2011). The resulting complex features two major AD risk factors in close proximity to each other and the site of a potential neurodegenerative process.

The second proposed binding partner is Aβ. It is well-established that ApoE influences Aβ biology, although the nature of this interaction is not fully understood in molecular terms (Jones et al., 2011; Verghese et al., 2013; Garai et al., 2014; Kanekiyo et al., 2014; Ghosh et al., 2019; Oh et al., 2020). Meticulous research from the Holtzman laboratory details the lack of a direct interaction. Another laboratory, however, reports that the interaction is Zinc dependent, and studies from the Holtzman laboratory do not report Zinc being in their assays. It has been shown that Aβ binds to receptors such as LDLR in the absence of ApoE, and it is known that ApoE facilitates the earliest steps in Aβ self-association as it forms large aggregates. The influence of ApoE may not be from direct physical contact with Aβ (Cho et al., 2001; Kara et al., 2018; Tsiolaki et al., 2019). A CR1+7S: C3b_2_: Factor I: ApoE: Aβ complex is entirely possible from the perspective of biophysical chemistry. Factor I has two LDLR domains. We suggest that ApoE binds one domain, and Aβ binds ApoE or the second LDLR domain, putting these two molecular entities in close proximity. As described above, we anticipate that Zinc is present under physiological stress conditions, but our model does not demand a molecular preference.

The sequence of the Aβ peptide shown here is littered with a mix of charged and hydrophobic residues and short amino acid repeats, such as HH, FF, and GG: DAEFRHDSGYEVHHQKLVFFAEDVGSNKGAIIGLMVGGVVIA.

This protein region is ambiguously structured before enzymatic cleavage from APP. However, once it is aggregated into amyloid, it is a highly structured β-sheet predominant sequence. Selected regions of the Sushi domains in CR1^2^ are also ambiguous with respect to secondary structure, and it has been proposed that these regions of intrinsic disorder can associate with other labile regions of similar ambiguous structure. These regions marked in Figure 4C are located at or near the bends in the CR1 atomic structure. One such segment is the bridge between Sushi 15 and Sushi 16. This region is within the C3b binding site and it is found in the repeated domains for this CR1 variant as well. This particular intermolecular interaction between two protein strains with intrinsic disorder is described as a “gel” where the interactions between the participating protein segments resemble the sticky interactions of two pieces of Velcro tape. This is because the interaction is strengthened by hydrophobic and electrostatic forces, but still reversible if the forces are counteracted.

If we focus on these Sushi domain elements that are intrinsically disordered to ask what role they might play, two prospects become apparent. First, these regions are analogous to the disordered Sushi domain in GABA_B_/a2 receptors that bind APP (Dinamarca et al., 2019; Rice et al., 2019), so APP may bind to this location. The binding site on APP itself has significant intrinsic disorder,^2^ in keeping with the nature of protein – protein association (Molliex et al., 2015; Banani et al., 2017; Hughes et al., 2018; Franzmann and Alberti, 2019) in this discussion. Two, monomeric or oligomeric Aβ may interact with these same regions of the CR1 receptor. Aβ, at the level of monomer or oligomer, is not yet a firmly and irrevocably stack of β-strands at this point in the process of self-association and the gel-like interaction is in keeping with a dynamic folding intermediate in transition. It is quite possible that this is an environment that is *permissive* for amyloid formation but only when factors such as Aβ concentration and solution conditions are favorable for this event. We suggest that this type of complex would provide a depot of soluble Aβ peptide still in reversible equilibrium, yet amyloidogenic under favorable conditions.

To put these processes in the simplest possible terms, consider an analogy: amyloid and the interstitial fluid/CSF compartments are like the oil and vinegar of a basic salad dressing. The oil and vinegar are not miscible except to the smallest extent. Something like a Caesar dressing is homogenous due to the presence of an emulsifying agent, which in our analogy is the ApoE: CR1 complex with the lipids of ApoE and the regions of both CR1 and Aβ with intrinsic disorder forming a gel-like interaction. This dynamic arrangement is metastable until perturbations, such as increased Aβ concentration, provide an alternative arrangement with greater stability.

**Figure 6:**
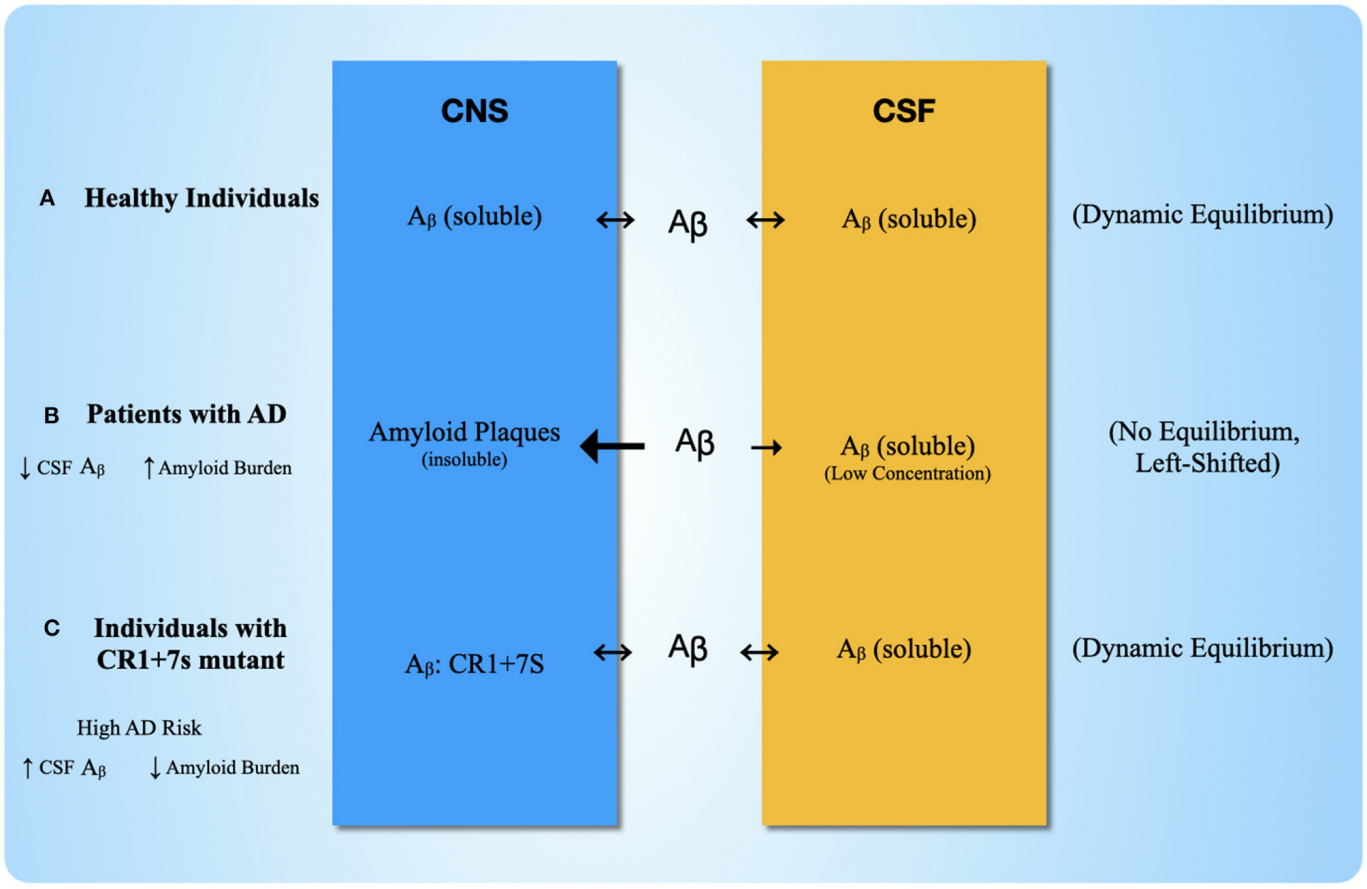
Plausible Explanation for Anomalous Clinical Data with CR1 with seven Sushi Duplication Chemical equilibrium for Aβ in three different scenarios. **(A)** In Scenario one, healthy individuals show a balanced chemical equilibrium, where Aβ moves into both the CNS extracellular space and the CSF at equal rates and remains soluble. **(B)** Scenario two shows the disruptions that occur to the equilibrium in AD patients. The Aβ in the CNS becomes insoluble in amyloid plaques, which shifts the equilibrium in favor of plaque formation in the CNS. The result is reduced levels of Aβ in the CSF. **(C)** Scenario three shows the equilibrium for individuals with the CR1+7s mutant. Aβ is able to bind CR1+7S, which serves as a depot for Aβ accumulation in the CNS but does not result in plaque formation, allowing continued equilibrium of Aβ into the CSF. Therefore, for those with the CR1+7S mutant, Aβ levels in the CSF will appear “normal,” but there is still an increased risk for neurodegeneration via the extra C3b binding site.

We have detailed the mechanics of this binding process in detail because such a complex would imply a potential explanation for an anomaly in clinical data (Schjeide et al., 2011; Brouwers et al., 2012; Thambisetty et al., 2013). Specifically, a complex of CR1+7S: ApoE: Aβ could resolve the apparent paradox of why patients with the CR1+7S variant show completely opposite amyloid distributions compared to AD patients, yet still exhibit increased AD risk. APP is cleaved to soluble Aβ_1−42_, which is an intermediate between APP and amyloid aggregate formation. Chemically, Aβ_1−42_ is in dynamic equilibrium between the CNS interstitial fluid and the cerebrospinal fluid (CSF). In AD patients, the Aβ_1−42_ becomes insoluble, creating amyloid plaques and altering the chemical equilibrium by the removal of soluble Aβ_1−42_. The resultant clinical finding for AD patients is high amyloid in the CNS and low Aβ_1−42_ in the CSF due to the redistribution of Aβ_1−42_ into amyloid. For people with the CR1+7S allele, however, they show the unusual finding of lower amyloid burden (by amyloid—PET scan) and a higher concentration of Aβ_1−42_ in the CSF (~20%), yet still demonstrate increased AD risk. If Aβ binds ApoE: CR1+7S, this provides a depot for Aβ that will continue to participate in the chemical equilibrium (Figure 6). Therefore, the Aβ: CR1+7S complex would explain the limited plaque formation and higher [Aβ_1−42_] in the CSF relative to AD patients, while still enabling destruction of host CNS tissue via C3b retention on host cell surface.

**Figure 7:**
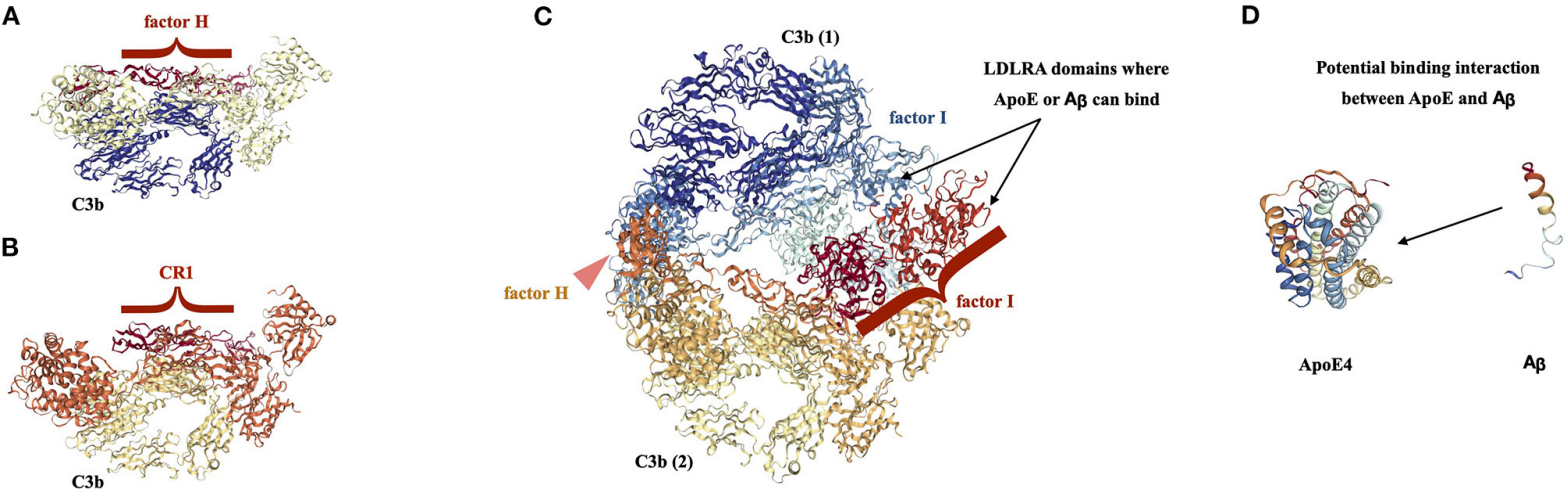
Proposed complex of CR1+7S with Additional Plasma Factors Associated with AD. Crystallographic structures from Protein Data Bank for selected RCA proteins in complex with Complement C3b. Original source for the crystallographic structures, Piet Gros laboratory (Wu et al., 2009; Forneris et al., 2016; Xue et al., 2016). These structures are used as a basis to suggest a macromolecular complex of the composition: CR1+7S: C3b: C3b: CR1+7S: ApoE. **(A)** Factor H: C3b complex; PDB structure, 2W11. This is a structure of the tub-like C3b structure in cream and navy, and the extended paddle-like structure of the four Sushi domains of factor H that bind to C3b resting on the lip of the tub, in brick red. **(B)** Complement Receptor 1: C3b complex, PDB structure 5FO9. This is a structure of the tub-like C3b structure in gold, and burnt orange, with the extended paddle—like structure of the four Sushi domains of Complement Receptor 1 that bind to C3b resting on the lip of the tub, in brick red. A comparison of the two C3b complexes where the binding partner is an RCA protein domain of consecutive Sushi domains yields many similarities. If one uses the base of the structure where there is a circular alpha helical tunnel as an orientation point, it can be seen that the C3b portion of the structures are similar to one another in pattern with similar layers of protein secondary structure. The upper surface of C3b is similar in each case and the Sushi domains of the RCA protein nestle within the middle of the structure in a nearly linear array of Sushi domains. **(C)** Factor H: C3b: Factor I complex; 5O32. This is a structure of two C3b domains, one in the lower half of the structure that is oriented analogously to the C3b subunits in 5a and 5b, colors yellow and gold, and a second C3b subunit in the upper half of the structure that is inverted, colors navy, slate blue. Within the middle of the C3b “sandwich” are both a Factor H Sushi string in burnt orange, and a Factor I subunit, in aqua and two shades of red. An analogous ternary structure for CR1, C3b, and factor I does not exist, but we believe that such a structure would be similar to this one, based on the extensive similarity of the Factor H: C3b and CR1: C3b structures that can be observed in **(A,B)**. We contend that this structure is informative about the arrangement of a mega complex that might include Aβ or APP binding to the Sushi domains of CR1, quite possibly at the newly formed Sushi – Sushi interfaces made due to the seven Sushi duplication and insertion, and Apoε binding to the LDLR-A domains of factor I, shown in the two shades of red in **(C)**. **(D)** ApoE and Aβ are two additional binding partners that may bind to the complex via the LDLR domains in factor I. Each of these factors may bind to an LDLR domain, or the complex of ApoE |Aβ may bind to an LDLR domain.

In sum, through the nuts and bolts of molecular recognition, it is plausible to conceive a mega-complex containing CR1+7S: C3b_2_: Factor I: ApoE: Aβ (Figure 7). This is the complex we propose; however, the CR1 is “shown” in the crystallographic image as factor H, because that is the published crystal structure for a ternary complex between an RCA protein, C3b and Factor I. Additionally, in the same figure, we show separate crystal structures for both a factor H: C3b complex and a CR1: C3b complex to show how greatly analogous these two structures are, due to the common domain arrangements of the RCA family proteins. We anticipate that the larger complex may be greatly analogous as well, with the CR1 domains simply replacing the factor H domains in the proposed complex.

What potential clues does a CR1+7S: C3b_2_: Factor I: ApoE: Aβ megacomplex give us about the disease process? Similar to detectives being able to place two high-profile suspects at the scene of the crime together, the significance of this complex is that ApoE and CR1, both strong AD risk factors, are now found together in a situation where neurodegenerative processes are possible. Our model is hypothetical, but this constellation of findings is chemically plausible and sits at the intersection of explaining several clinical findings in AD. Additionally, it nests nicely with two discussions of this phenomenon already in the literature: the “vanishing” amyloid by Gandy et al. (2013) and a hypothesized sequential relationship between C3, ApoE, amyloid, and tau (Bonham et al., 2016). Furthermore, this hypothesis is testable by straightforward methods.

### Other Considerations: Cell Surface Sialic Acid

Though not formally considered a component of the complement pathway, sialic acid is noteworthy as a renewed area of scientific interest that fits the theme of complement dysregulation. Sialic acid is involved in regulating complement activity and, in the brain, microglia activity. Sialic acid offers a sanctuary for soluble RCA proteins such as Factor H to dock on the membrane surface. This provides a high local concentration of soluble RCA protein in a conformationally adapted form to be at the ready for cell surface complement regulation (Ferreira et al., 2010; Schmidt et al., 2018). In this way, sialic acid contributes to timely regulation of complement activity in the periphery when all complement components other than the cell bound RCA proteins are soluble and found in ~3 L of blood plasma, due to local concentration effects.

It is not clear that the value is the same in the tightly packed space of the CNS, where there may be an emphasis on local complement synthesis instead (Blaum et al., 2015). Microglia contain surface sialic acid and this moiety is tightly regulated. It has been shown that sialic acid can attenuate the extent of inflammatory damage inflicted by microglia involvement (Griciuc et al., 2013; Miles et al., 2019; Yang et al., 2019; Puigdellivol et al., 2020). If sialic acid is abundant, microglia are not activated toward phagocytosis. In contrast, if the sialic acid has been enzymatically removed, activated microglia aggressively phagocytose. This activation is controlled by microglia cell surface receptor CD33—also a risk gene for AD—and is an early step in the recruitment of microglia from routine surveillance to active engulfment of proximal matter. These features imply a concept for immune dysregulation via sialic acid, and this is an active area of investigation as a CNS therapeutic target. However, the jury is still out on the magnitude of effect such a process might have. For instance, neurons have a limited amount of surface sialic acid and this amount is not altered in AD (Griciuc et al., 2013). We believe this cell signaling dynamic merits further study, but we estimate that the impact to intact neuronal circuitry might be less than other changes discussed here. Thus, in our model, we focus on direct factors that impact synaptic integrity.

## Overwhelmed C1-Inhibitor Leads to Overactive Complement

### At What Level Is C1-INH “Overwhelmed?”

The enzymes of Complement C1 are active for only a short time before they are irreversibly inhibited by C1 Inhibitor (C1-INH), which circulates freely in biological fluids (Ziccardi and Cooper, 1979; Ziccardi, 1981, 1982; Windfuhr et al., 2005). Upon recognition of an activated C1 enzyme, C1-INH covalently attaches to the active site, preventing further activity of C1 and, consequently, the entire pathway. C1-INH activity is stoichiometric: one C1-INH molecule is used for each and every molecule of enzyme that is blocked.

C1-INH has multiple targets other than C1. Most significant for this discussion are enzymes of the contact activation system: kallikrein, Factor XI and Factor XII (Davis et al., 2008; Conway, 2015; Yang et al., 2016). The contact activation system is a four-step pathway that promotes blood coagulation, and there are conditions where both classical complement and contact pathway activation are taking place at the same time. Under these circumstances, C1-INH availability may become a factor in adequate regulation for cessation of enzyme activity. This concept is clearly illustrated by hereditary angioneurotic edema (HAE, specifically HAE Type I, HAE Type III), where a single dysfunctional C1-INH allele (resulting in depleted C1-INH levels) culminates in potentially life-threatening levels of vasopermeability (Späth et al., 1984; Cugno et al., 2009; Hack et al., 2012; Schmaier, 2019; Kajdacsi et al., 2020).

The average C1-INH plasma level in HAE is ~40%% normal (Yang et al., 2016; Castellano et al., 2018; Cicardi and Zuraw, 2018; De Maat et al., 2018), yet this is sufficient to provide normal immune function on most days. During an attack of angioedema, the C1-INH levels drop to 10–20% normal through consumption of the C1-INH in its role to inhibit plasma serine proteases. Table 1 lists the normal levels of all plasma serine proteases that are inhibited by C1-INH. C1-INH is not at molar excess to any of the pathways it inhibits. Since inhibition is by formation of a 1:1 complex between C1-INH and the protease, the C1-INH protein is insufficient to inhibit all of the enzymes in any one of the pathways, particularly the contact activation cluster of enzymes. That said, the pertinent biology for both complement and contact activation is local activation and inhibition over a small anatomical area at any one time, for pathogen clearance or localized vasopermeability and these events occur as necessary. Using HAE as a blueprint, we can establish a lower limit for effective C1-INH concentration at ~0.06 umole/L, or ~40% normal value.

**Table 1:** Stoichiometry of C1-inhibitor and the enzymes it inhibits.

**C1-INH**		**Enzymes Inhibited by C1-INH**							
		**Classical pathway**		**Contact pathway**			**Lectin pathway**		
Resting	Acute Phase	C1r	C1s	Kallikrein	Factor XI	Factor XII	MASP I	MASP II	MASP III
0.16 μmole/L	0.35 μmole/L	0.2 μmole/L	0.2 μmole/L	0.1 μmole/L	30 nm/L	0.5 μmole/L	70 nm/L	5 nm/L	0.2 μmole/L
Total: 0.16–0.35 μmole/L		Total: 0.4 μmole/L		Total: 0.63 μmole/L			Total: 0.275 μmole/L		
**0.16–0.35** **μmole/L** (Total C1-INH)		**1.305** **μmole/L** (Total of all inhibited enzymes)							

### C1 INH Insufficiency in AD

Lack of complement regulation in AD has been documented since 1999. The McGeer laboratory (Yasojima et al., 1999) found that C1-INH was disproportionately distributed in brain areas of active AD. Compared to normal tissue, complement levels were increased to ~3 times for early pathway components (e.g., C1) and up to 20 times for the cytolytic MAC complex. In contrast, C1-INH levels were only increased by ~2 times. The previous section showed that C1-INH is sub-stoichiometric to the pathways it inhibits, and this experiment provides a clear picture within AD where C1-INH levels did not match increased complement activation.

We suggest that AD may have resemblance to an HAE attack in the sense of dysregulation from C1-INH depletion to < ~40% normal value. In our model, the CNS classical complement cascade participates in its housekeeping role up to the point of C1-INH depletion. After that point, dysregulated complement activation and contact pathway activation may be features of AD due to the unregulated level of activation in both pathways. The concept applies, since neuroinflammation is a major aspect of AD (Späth et al., 1984; Conway, 2015; Yang et al., 2016), as is neurodegeneration, which we propose here may be related to haywire complement activity. Two possible scenarios might lead to C1-INH depletion: (1) a situation of degenerative pathology that overwhelms the clearance capacity for complement, such that what would normally be a brief pulse of complement activity becomes a continuous event over repetitive attempts to clear an insurmountable quantity of unwanted material, and (2) simultaneous activity of complement activation and contact pathway activation.

The first scenario is immanently plausible: this could be as simple as complement becoming overwhelmed by the continuous accumulation of both amyloid and NfT, along with attempts to clear damaged synapses. It is established that complement binds amyloid, NfT, and synapses. Likewise, the progression of pathology in AD is well-documented. Self-association of Aβ fragments into oligomers and fibrils is a necessary, but not sufficient, first step. Next, the hyperphosphorylation of Tau protein is followed by tau aggregation into neurofibrillary tangles. NfT formation tracks in a synchronized manner with cognitive impairment, which is caused by synapse damage and loss, so complement-mediated synapse clearance must begin prior to, or simultaneous with tau pathology. At the inception of mild cognitive impairment, or perhaps just before, these three processes are simultaneously ongoing. Each of these processes involves complement activation and clearance, and thereby, complement regulation by C1-INH and the RCA protein family.

So far, the study of C1-INH in AD has not been approached from this type of mechanistic perspective. It has, however, been investigated as a possible biomarker in several clinical trials on AD. From these reports, we know that C1-INH levels are insufficient at the time of the diagnosis of mild cognitive impairment. This finding is consistent with early complement measurements in AD where it was reported that the levels of C1-INH did not increase to the same extent as the pathway components, such as C3 and complement proteins of the membrane attack complex, C5-C9 (Yasojima et al., 1999). Regarding C1-INH reported values at the time of mild cognitive impairment, the study design/methodologies vary and the conclusions are mixed. Three separate studies representing a total of ~500 patients with mild cognitive impairment or worse clinical picture report a reduced C1-INH level to ~60% normal (Cutler et al., 2008; Muenchhoff et al., 2015; Shen et al., 2017; Singh et al., 2020). Indeed, one of these studies from the Strickland laboratory at The Rockefeller University, discussed below, is a combined study where the biological consequences of C1-INH depletion are documented as well. Other studies simply report C1-INH levels in a panel of putative biomarkers to corroborate with a diagnosis of mild cognitive impairment, where there is a distinct correlation.

At this time, one can only speculate about the fate of the missing 40% C1-INH. Fortunately, AD patients are not prone to unexpected bouts of edema as the level is not in the HAE crisis range, and it is not expected that there is significant complement or contact activation going on in the periphery. This leads us to the prediction that the C1-INH has been consumed at the BBB or within the CNS. The translocation of C1-INH to the brain has been observed in a C1-INH knockout mouse, strengthening this position (Farfara et al., 2019).

The second scenario—simultaneous activation of the CCP and contact pathways—is verifiable by straightforward research but the question has not been addressed directly. The remainder of this section will explore the current literature supporting this concept.

There is abundant evidence for contact pathway activation in AD (Shibiyama et al., 1999; Bergamschini et al., 2001; Maas et al., 2008; Strickland, 2018). Cleaved factor XII, an indicator of contact activation, has been measured in plasma from AD patients in separate collections and from separate laboratories (Zamolodchikov et al., 2015; Suidan et al., 2018). Cleaved factor XII has been found in AD plasma to a greater extent than normal plasma from age–matched controls. Additionally, Factor XII KO (by anti-sense technology) mice have been used to evaluate the effect of reduced contact pathway activity on AD. These mice showed less microglia activation, reduced neuron damage, reduced cognitive impairment, and less overall brain damage (Chen et al., 2017). Bradykinin is also an indicator of contact pathway activation. In the periphery, Cicardi et al. showed that the life-threatening inflammation of HAE is driven by excess bradykinin levels (Curd and Prograis, 1980; Nussberger et al., 1998, 1999). In the CNS, Strickland et al. measured plasma clotting times and bradykinin levels (plasma and CSF) in AD patients directly (Bergamschini et al., 2001), finding that the level of bradykinin increased linearly with the severity of cognitive impairment and the AD biomarkers, neurofilament light chain and Aβ_1−42_. This clearly showed a direct relationship between contact activation and the severity of AD. Together, these two studies show that contact pathway activation tracks with harmful inflammation in HAE and AD, with HAE being a known disease of C1-INH depletion. This reinforce our hypothesis that excess contact activation in AD is linked to C1- INH depletion.

C1-INH KO mice (antisense technology) also show relevant vascular features. A C1-INH KO mouse model was found to have significant immune reactivity, including IL-6 release and induction of nitric oxide synthase, indicating considerable CNS inflammation (Farfara et al., 2019). Moreover, the compromised BBB was accompanied by infiltration of immune reactive cells from the periphery. The fact that C1-INH depletion is linked to BBB compromise lends further support to our model: if there is an occasion where continuous complement and contact pathway activity is ongoing, it is likely at or near the BBB. This is significant because there is a strong correlation between vascular dementia and AD. In some cases, vascular dementia has been shown as a precursor to AD dementia (De la Torre, 2004; Zlokovic, 2011; Iadecola, 2013; Kisler et al., 2017), though this is not the universal case.

Compromised integrity of the BBB is known to be a feature of AD, but at what point in the disease process it is compromised is not fully understood. For carriers of the Apoε4 allele, deterioration of the BBB occurs earlier (Montagne et al., 2020). A recent study of the timeline from young to aged mice described fundamental changes to the exchange process at the BBB endothelial cell surface (Yang et al., 2020). In younger animals, exchange of blood components was tightly controlled by receptor-mediated endocytosis via clathrin coated pits. In contrast, older animals showed loss of pericytes and CNS protection in the extraluminal space. These changes were accompanied by a shift to generalized endocytosis with less chemical specificity, such that the BBB was now permeable to a wider range of macromolecules. A proteome-based study of the early cortical lesions in multiple sclerosis found evidence for contact activation, complement activation, and iron dysregulation (Magliozi et al., 2019), so it is quite possible this same constellation is an aspect of early-stage AD as well.

Autopsy samples demonstrate that microvascular compromise with pericyte damage and loss of function is present in AD (Zenaro et al., 2017; Nation et al., 2019). A common condition found in small arteries is cerebral amyloid angiopathy (CAA) (Carriano et al., 2012; Halliday et al., 2016; Hernandez et al., 2019), which is more prevalent in carriers of the Apoε4 allele (Hultman et al., 2013). CAA is identified by the presence of amyloid within the walls of small-diameter arteries in the brain, and the amyloid typically is Aβ-based but not always Aβ-derived. The aggregates are known to activate the complement pathway (Matsuo et al., 2017) and introduce a coagulopathy, likely based on the presence of a consequential irregular surface to the vessel wall. Contact activation is one aspect of the coagulopathy (Nishitsuji et al., 2011), so the only unknown component to our suggestion is iron dysregulation. From the groundbreaking work of Katerina Akassoglou's laboratory, an early, partial list of proteins that enter into the brain parenchyma has been reported from work on MS (Bardehle et al., 2015; Merlini et al., 2019; Akassaglou, 2020). Among these is the clotting protein fibrinogen. With the expectation that there is a certain commonality to early degeneration across several diseases, it is well-worth a similar study in AD. Using an integrated approach that included bioinformatics and mRNA screening of brain samples subjected to oxidative stress, Dr. Akassaglou and colleagues have confirmed the significance of glutathione metabolism in early stages of MS (Mendiola et al., 2020). Glutathione dysregulation is a hallmark of the iron-mediated cell death known as ferroptosis, suggesting the findings concur with the proteomic analysis.

In summary, simultaneous activation of the complement and contact pathways could overwhelm C1-INH to a dangerous level. This idea has not been tested directly; however, the results of several independent studies consistently support the concept of C1-INH as a gatekeeper for immune activity and inflammation. We recommend this as a topic of potentially high-yield investigation. The exciting implication is that, if confirmed that C1-INH depletion is promoting the progression of AD, C1-INH replacement would be a potential neurotherapeutic agent.

### Theoretical Applications for the Ratio of Complement to Inhibitors of Complement

Within the framework of our model, insufficient or dysregulated complement regulators would influence the amount of excess, “bystander” synaptic pruning from inappropriate complement activity and/or inflammatory processes underway in the CNS. If validated, this concept has potential application to companion biomarkers. The relative values of regulatory molecules (such as C1-INH) and available complement or consumed complement would give a readout about the effective regulation of complement. If the level of complement regulators is withing working range, it can be expected that one is protected by the appropriate level of complement providing protection as part of the innate immune system. If the levels are not appropriate, this finding would provide an alarm for the possible cell and tissue destruction that is ongoing instead.

## CSMD Proteins Are Putative Complement Control Proteins

### CSMD1 Sushi Domains 17-21 Mimic C4bp to Limit C3b Formation

In the brain, the Cub Sushi Multiple Domain (CSMD) proteins are located at the post-synaptic density (PSD), a thickened membrane segment with an assembly of scaffolding molecules that anchor neurotransmitter receptors and participate in the signaling that follows neurotransmitter binding (Kraus et al., 2006; Havik et al., 2011; Steen et al., 2013; Mizukami et al., 2016; Athanasiu et al., 2017; Gutierrez et al., 2019). These CSMD proteins are known loci for disease susceptibility. CSMD1 knock-out (KO) mice have increased anxiety, reduced cognition, and additional mood disturbances (Kraus et al., 2006; Havik et al., 2011; Steen et al., 2013; Athanasiu et al., 2017). CSMD2 is a schizophrenia risk gene and KO animals have reduced cognition (Havik et al., 2011; Athanasiu et al., 2017; Gutierrez et al., 2019). CSMD3 can be found on dendrites of hippocampal neurons. It is thought to play a role in dendrite development, and CSMD3 overexpression has been correlated to increased branching (Mizukami et al., 2016).

CSMD proteins are putative complement control proteins, based on domain homologies to proteins in the RCA cluster and complement pathway enzymes. The domain arrangement of these proteins is similar to one another and unusual in that the Sushi domains are both interspersed with the CUB domains and in a separate sequential string, similar to the RCA cluster of complement control proteins. In a laboratory evaluation of complement function, a soluble CSMD1-like construct of CUB/Sushi/Sushi12 caused reduced C3b deposition on a cell surface where complement activation was measured (Escuerdo-Esparaza et al., 2013). This was explained by the later finding that Sushi domains 17–21 of CSMD1 mimic complement component C4bp (Magdalon et al., 2020), inhibiting the classical C3 convertase in a dose-dependent manner. Therefore, CSMD1 provides a regulatory stop signal for C3b generation and any further clearance from the classical complement pathway. It is located uniquely at the PSD where synaptic pruning might be “in order,” but still needs to be carried out in a manner that is carefully regulated.

### CSMD Proteins May Participate in a Cell Signaling Complex for Synapse Formation and Dendrite Expansion

Do the unique CUB/Sushi domains of CSMD proteins have a separate function that is distinct from the regulation of complement activity? It is not known. From the perspective of protein biochemistry, however, the question is certainly worth asking. The string of Sushi domains in these proteins is analogous to the domain arrangement of the RCA proteins. However, none of the RCA proteins have a string of Sushi domains in the alternating CUB | Sushi register that is found in CSMD proteins. The section above presents both structural and functional evidence for CSMD proteins to function as complement control proteins similar to the RCA proteins. The discussion that follows is more speculative: we draw on biophysical relationships to highlight the suggestion of a second CSMD function consistent with the theme of complement biology.

The sequential CUB/Sushi cluster is found in several serine proteases within the complement family (Wallis, 2007; Gaboriaud et al., 2014). The CUB domain is most often an interaction domain, not the enzymatic domain. For many complement enzymes the CUB domain heavy chain creates substrate exclusivity for the generic serine protease active site (Gaboriaud et al., 2014). CUB domains are found in other enzymes as well, such as metalloproteinases and phosphatases (Gaboriaud et al., 2011), both commonly found in cell signaling complexes and membrane remodeling enzymes. Typically, metalloproteinases are membranous enzymes that digest extracellular matrix proteins as one aspect of tissue remodeling and phosphatases participate in cell signaling biology directed at tissue growth. As one example, CUB-domain containing protein 1 (CDCP1) promotes matrix metalloproteinase-9 secretion and degradation of extracellular matrix (Miyazawa et al., 2010).

**Figure 8:**
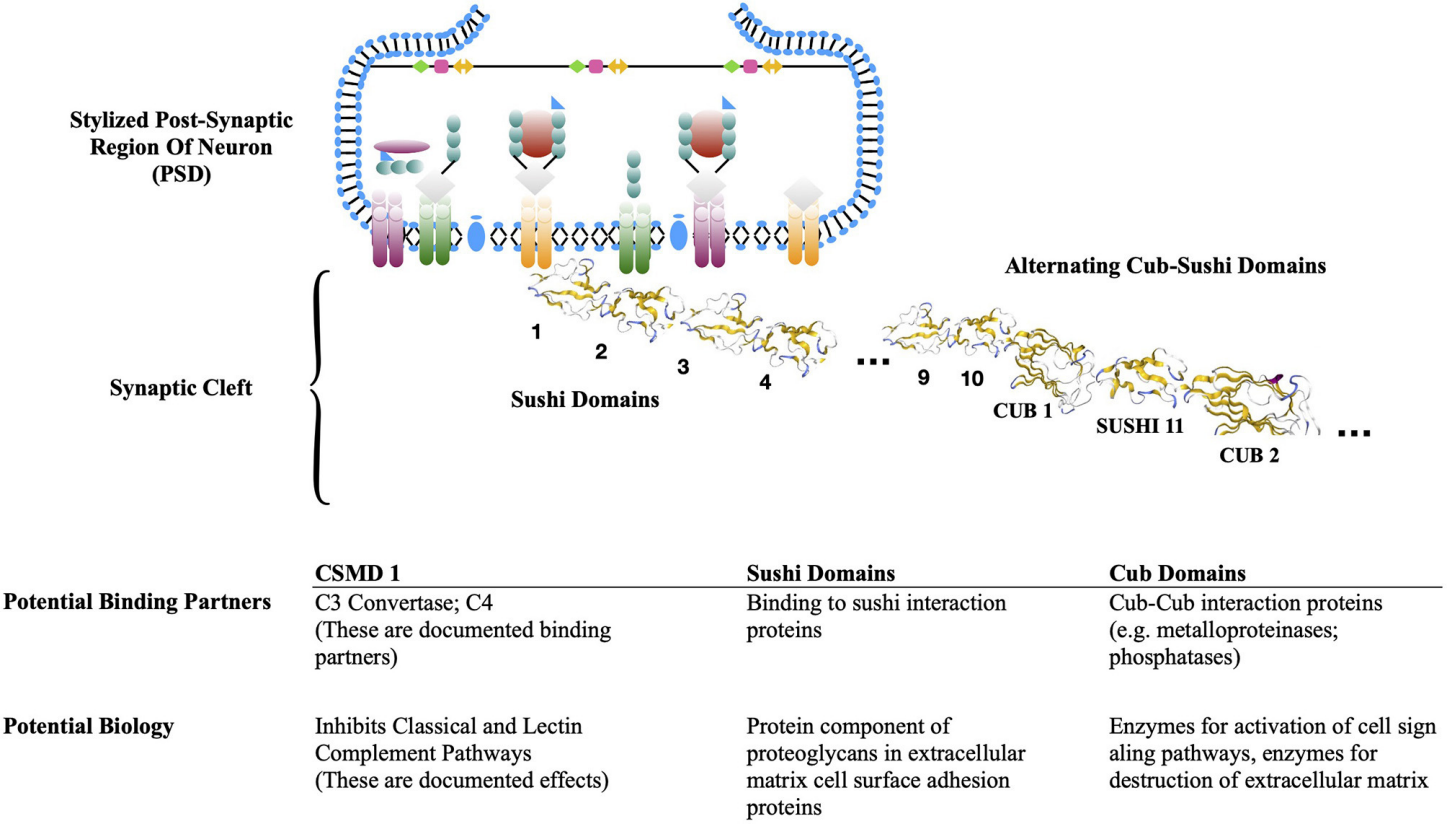
Post-synaptic density with CSMD. Model of CSMD protein at the protein-rich PSD. CSMD proteins are membrane bound proteins with a transmembrane domain and an extensive extracellular domain with a defined structure of 10–14 consecutive Sushi domains, then alternating CUB-Sushi domains. A stylized PSD and a CSMD protein with representative CUB and Sushi crystal structure are shown. The table below describes anticipated binding partners based on the families of proteins with CUB domains and/or Sushi domains.

Sushi domains are found in many proteins as well, many of which are binding partners to the protein portion of proteoglycans on the cell surface and integral to the extracellular matrix (Makou et al., 2015). Going by structural homology and location, we speculate that the CSMD proteins could be part of cell signaling centers involved in the essentials of new synapse formation and possibly dendrite growth and expansion (Figure 8). What might that signal be, and is it related to complement activity? Previous literature is sparse. A recent paper from Molofsky et al. describes crosstalk between the neuron and microglia via neuronal synthesis of IL33 and microglia expression of an IL33 receptor that mediates microglia-driven engulfment of extracellular matrix components (Nguyen et al., 2020). We hypothesize that a CSMD signaling complex might be one aspect of this process to make room for new synapses. Since the PSD is where C1q has been visualized (Dejanovic et al., 2018; Litvinchuk et al., 2018), it is plausible that C1q or C1 binds to the CSMD complex via a CUB domain(s) to initiate either clearance via traditional complement steps or recovery through a cell signaling mechanism within the CSMD complex. Other models are possible, and the C-terminal section of sequential Sushi domains may be the location of binding partners for another purpose.

Given a putative location of CSMD1 at the PSD, this fits the concept of CSMD proteins acting within a cell-signaling complex directed at synaptic and/or dendritic growth, where inhibition of bystander complement activity would be of paramount importance. This can also be investigated experimentally. As one example, it would be informative to compare synapses and wild type and CSMD KO for synaptic density, dendrite patterns, and factors associated with synapse and dendrite formation such as metalloproteinases and phosphatases. If this hypothesis is born out, CSMD proteins provide an essential biological switch aimed at synaptic retention and synaptic growth, two essential features of robust neuronal circuitry required for learning and memory.

## Conclusions

We have detailed how perturbations of complement regulation may disrupt neuronal homeostasis within the brain. Combining complement biochemistry with advances in neuroscience, we offer the perspective that complement may be a double-edged sword: improperly regulated, the blade may just as swiftly damage the body it is meant to defend. Regulatory features such as C1-Inhibitor have not been explored, and proteins of the RCA cluster have not been viewed in their proper protective role.

Disabled proteins within the RCA cluster, such as Factor H, result in C3b-mediated tissue damage in the periphery. Application of this concept to the CNS is straightforward and merits experimental investigation. While we know the classical complement pathway proceeds up to C3 cleavage within the CNS, complete complement factor analysis is missing and would greatly illuminate this area of biology. The most compelling example is CR1, which is among the strongest AD risk factors. Some regions of CR1 are intolerant of even minor point mutations, and since very few molecules of CR1 are present on the cell surface, even a small disruption to CR1 function or expression could impact its ability to clear C3b.Additionally, from a protein domain perspective, the CR1+7S variant not only explains clinical findings for varying levels of amyloid burden, but also provides a mechanism for how CR1 and ApoE may interact with one another at the site of a neurodegenerative process.

C1-Inhibitor suppresses excess activation of complement and contact activation. Should this vital regulator be depleted or overwhelmed, the destructive power of complement is free to be unleashed on the body and brain with devastating effect. This is known beyond doubt in hereditary angioneurotic edema, and we have outlined how this may be a compelling explanation for the neurodegeneration in AD as well. The exciting consequence of this model is that, if confirmed, C1-INH replacement would be implicated as a viable neurotherapeutic intervention. Moreover, the concept implies measurements of complement inhibitor availability could comprise at least two sets of biomarkers to assess how well-complement regulation is working.

Finally, the CSMD proteins are known loci of disease susceptibility in the brain and their depletion creates behavioral changes in mice. We have outlined how they may function as putative complement control proteins in similar fashion to the RCA family of proteins. Additionally, given their location at the PSD and unique domain composition, we have put forward an intriguing position regarding these proteins as integral features of cell-signaling centers that control synapse retention and new synapse formation and dendrite growth. By structural comparison, metalloproteinases, and phosphatases are possible ligands. This putative function links complement to multiple CNS disease loci and is based on testable biochemical models.

Our model is hypothetical and must be understood as such. We have aimed to illustrate the merit of complement dsyregulation in the CNS as a timely and worthwhile object of study and attempted to provoke new ways of thinking about age-old processes. Furthermore, we have striven to highlight ideas that are testable within the current framework of knowledge, including: (1) the CR1 megacomplex, (2) molecular dissection of cells to understand what complexes are present, (3) complement factor analysis via immunohistochemical studies, (4) the use of complement measurements as companion biomarkers for disease and CNS status, to provide an early picture of neurodegeneration, (5) the use of complement measurements to evaluate the extent of inflammation, and (6) comparing wild type and CSMD KO synapses for patterns of factors associated with dendritic and synaptic growth. Our major ideas–including the analogy of HAE to C1-INH insufficiency in the CNS, CR1 and apoE as factors in AD, plus Factor H in ocular and renal disease – are grounded in human studies. In most cases, we limited our use of publications with animal data to literature where the essential points of the manuscript were confirmed with the analysis of relevant human samples.

In sum, regulated complement is an integral aspect of optimal brain function and development, while (as we have argued) dysregulation contributes to disease. Viewing CNS pathology through this lens generates new questions and rich potential for experimental and theoretical progress. Neuro-immunology is in its infancy and complement regulation will be a defining feature as this field comes of age. Regardless of whether the particulars of this model are ultimately confirmed or falsified, understanding the role of complement regulation in the CNS—and how regulatory failure may produce disease states—is critical to expanding our study of intractable neurodegenerative conditions such as Alzheimer's. This manuscript is therefore a call to action for research to clarify the transition from normal physiology to pathophysiology that describes the early stages of disease. We believe this will open new diagnostic and therapeutic options for CNS disease.

## Data Availability Statement

The original contributions presented in the study are included in the article/supplementary material, further inquiries can be directed to the corresponding author/s.

## Author's Note

We dedicate this article to Marco Cicardi, MD, and Robert David Moir, PhD. Both these excellent scientists left this world too soon, but their quality contributions to medical research are long-lasting. It is up to those who remain to carry their quality legacy forward.

## Author Contributions

CS conceived the concept of immune dysregulation and linkage between HAE and AD. NP contributed interpretation of clinical data and clinical reviews. All authors wrote the manuscript, synthesized the figures, and approved the final manuscript composition.

## Conflict of Interest

CS is an unpaid consultant to IPPIN Biomarkers and will become a shareholder of the C-Corporation should it obtain Series A financial backing. The remaining author declares that the research was conducted in the absence of any commercial or financial relationships that could be construed as a potential conflict of interest.

## Footnotes

^1^In the literature, this mutant is often denoted as CR1-S or CR1^*^2.

^2^This mutation has a new Sushi-Sushi junction that is Sushi17 – Sushi 11 when the tandem duplication is created. Furthermore, if the CR1 amino acid sequence is examined by programs that predict intrinsic disorder in protein structure (Unipred, Pondr), the N-terminal amino acids in the region of the ~15th–20th amino acids are predicted to have intrinsic disorder in a selected number of the Sushi domains meaning that the structure is dynamically unstable and prone to association with like regions in other domains or other proteins.
